# Acute and Post-traumatic Stress Disorder Symptoms in Mothers and Fathers Following Childbirth: A Prospective Cohort Study

**DOI:** 10.3389/fpsyt.2020.562054

**Published:** 2020-12-22

**Authors:** Elisabeth Schobinger, Suzannah Stuijfzand, Antje Horsch

**Affiliations:** ^1^Institute of higher Education and Research in Healthcare in French, University of Lausanne, Lausanne, Switzerland; ^2^Department Woman-Mother-Child, Lausanne University Hospital, Lausanne, Switzerland

**Keywords:** acute stress (disorder), PTSD-post-traumatic stress disorder, parents, mothers, fathers, nurses and midwives, childbirth

## Abstract

**Introduction:**

Up to 30% of women view their childbirth as traumatic. This experience can lead to acute stress disorder or post-traumatic stress disorder. The negative impact of maternal post-traumatic stress disorder following childbirth reaches beyond the mother, potentially affecting her child's development and the couple's relationship. Research on *paternal* post-traumatic stress disorder following childbirth is scarce. Acute stress disorder is suggested to be an important predictor of post-traumatic stress disorder in mothers, but little is known about *paternal* acute stress disorder following childbirth. Furthermore, there is limited information about the comparison or relation of acute stress disorder and post-traumatic stress disorder following childbirth between parents.

**Aim:**

[1] To compare the prevalence rates and severity of acute stress disorder and post-traumatic stress disorder symptoms between parents following childbirth by taking anxiety and depression symptoms, as well as obstetric variables and previous traumatic events into account and [2] To determine if acute stress disorder is a predictor of post-traumatic stress disorder.

**Method:**

A prospective population-based design was used. *N* = 647 participants were recruited from future parents who attended appointments at the Obstetrics and Gynecology unit at a Swiss university hospital. Self-report questionnaires were used: Post-traumatic Diagnostic Scale in the third trimester of pregnancy (T1) and 1 month post-partum (T3), Acute Stress Disorder Scale at 1 week post-partum (T2), and Hospital Anxiety and Depression Scale at all time points. Obstetric and neonatal variables were retrieved from hospital records.

**Results:**

At T2, 8.9% of mothers and 4.4% of fathers presented symptoms of acute stress disorder. At T3, 20.7% of mothers and 7.2% of fathers had symptoms of post-traumatic stress disorder. Acute stress disorder was a predictor of post-partum post-traumatic stress disorder (Odds ratio: 8.6, IC 95% [1.85; 40.42]). Depression symptoms was a significant confounder in the prediction of post-traumatic stress disorder following childbirth, but not anxiety or previous perinatal loss.

**Conclusion:**

Little is known about parental differences in acute stress disorder and post-traumatic stress disorder symptoms following childbirth. Results indicate that *both* parents may suffer from acute stress disorder and post-traumatic stress disorder symptoms after childbirth and that acute stress disorder is a predictor of post-traumatic stress disorder after childbirth for both parents. Sensitization of maternity staff to these results may assist in earlier identification of and appropriate treatment for at-risk parents.

## Introduction

Childbirth is often experienced as a happy event, but up to one third of women view their childbirth as traumatic, which can lead to different psychological problems like depression, acute stress disorder (ASD) or post-traumatic stress disorder (PTSD) (1). ASD and PTSD are both trauma- and stressor-related disorders (2). ASD may appear after 3 days and lasts up to 1 month; if symptoms persist, then a diagnosis of PTSD becomes likely. ASD is thought to be a predictor of PTSD (3, 4). Both share the following symptom clusters: re-experiencing, avoidance, negative cognitions and mood, but ASD has one more cluster: dissociation (2).

For trauma-related symptoms occurring up to 1 month following traumatic childbirth, the diagnosis of ASD is given. ASD corresponds to PTSD in terms of symptoms, with some variations. ASD requires at least nine symptoms of any clusters cited above. If the symptoms last beyond 1 month, then a diagnosis of PTSD is relevant (2). PTSD following childbirth (PTSD-CB) is highly comorbid with depression (5–7). Furthermore, of those with PTSD-CB, 24% of mothers present symptoms of anxiety during pregnancy, whereas the fathers' rate tends to be lower (7–9).

PTSD according to DSM-5 (2) occurs after experiencing or witnessing an event that induced a threat of death or death, a severe injury, or physical threat. During childbirth, some women may experience an actual or perceived threat to their physical integrity, to their life and/or the child's (1). Symptoms of PTSD are grouped in four clusters: re-experiencing, avoidance, negative cognitions and mood, and hyperarousal. All of these symptoms must be present for at least 1 month and impede both social and professional life (2).

PTSD-CB has been widely studied in mothers after childbirth. Its prevalence is between three and six percent in community samples (without any complications/interventions) and increases to 18.5% in high risk samples (e.g., cesarean section) (5, 10, 11). ASD following childbirth (ASD-CB) has been less studied and prevalence rates of around 5.6% have been found (12).

PTSD-CB can impact negatively not only on the mother but also on the baby and the couple (10, 13, 14). Mothers can have negative feelings toward themselves (15, 16) and they may alter their family planning (17). Maternal PTSD-CB has also been associated with difficulties in establishing the mother-baby bond (18, 19), may impact breastfeeding initiation and continuation (20, 21) and negatively impact the child's sleep and development (13, 22). Perinatal mental health problems like post-partum PTSD have important economic costs, representing £8.1 billion in 2014 in the United Kingdom. The majority of those costs are related to the child, clearly showing the negative impact on the future generation (23).

Since the 1960's, fathers tend to be more present during labor and delivery, thus potentially increasing their risk of PTSD-CB (24, 25). Research on paternal PTSD-CB, however, is scarce. Paternal PTSD-CB incidence is between 0 and 5% (25–31). These estimates are based on the results of four studies using small samples size and cross-sectional designs at different times of measurement. Fathers' PTSD-CB has been studied in other contexts, like the Neonatal Intensive Care Unit (NICU), where the prevalence tends to be higher: from eight to 66% (4, 32–37). To our knowledge, ASD-CB has never been studied in fathers after childbirth. Given the multiple negative consequences of ASD-CB and PTSD-CB on the whole family, it is primordial that healthcare professionals be sensitive to this topic.

Fathers' ASD, to our knowledge, has not been studied after childbirth in the general population. Following the admission of their child to the NICU, fathers' ASD prevalence is between 20 and 28% (35, 38, 39). Studies on both parents' ASD in the pediatric intensive care unit (PICU) and NICU (35, 40) have found that ASD is a predictor of PTSD. Results show that 70% of parents suffering from ASD will suffer from PTSD (41), whereas 90% of parents who have no ASD will not develop PTSD (36, 42). Other studies in the general population have found that ASD is not specific enough to predict PTSD (43–45) and that ASD is a greater predictor of PTSD in women than men (46).

The lifetime risk for women to develop ASD and PTSD is two or three times higher than men (47). This is also supported by studies in the NICU comparing rates of parental ASD (35, 38, 48). ASD and PTSD also seem to present themselves differently according to gender. Mothers have been shown to have a higher severity score of ASD or PTSD than fathers (38, 49). Some studies have found that mothers have more intrusion, avoidance and hypervigilance symptoms than fathers (33, 50), although others found no symptom difference between parents (26, 48).

Although fathers seem to have less risk and symptoms, they remain important to the study of PTSD-CB. Fathers are the main support of their spouse after birth (27). They often put their spouse's needs first and tend to underreport difficulties (35, 51). It is also likely that a correlation between both parents' mental health status exists and that the immediate emotional state of the father in the post-partum might affect the mother's (26, 52, 53).

The aim of the study was firstly to compare the prevalence rates and severity of ASD-CB and PTSD-CB symptoms between parents following childbirth by taking anxiety and depression symptoms, as well as obstetric variables and previous traumatic events into account and secondly to determine if ASD-CB is a predictor of PTSD-CB.

The choice of study variables (and confounding variables in particular) is based on the diathesis-stress model of the etiology of PTSD-CB (54). The model of the etiology of PTSD-CB highlights the vulnerability and risks factors that can be divided into three categories: prepartum, peri-partum, and post-partum (54). Prepartum risk factors found to be significant are, previous traumatic birth or previous perinatal loss and previous mental health factors (54, 55). Some studies have found inconsistent results regarding sociodemographic factors, such as age or socio-economic status (55). Prepartum factors relevant for this study were: age, education, previous perinatal loss or traumatic birth, and psychological problems during pregnancy, such as depression and anxiety. A peripartum risk factor relevant here is operative birth (OB) (18, 56). Post-partum risk factors retained in our study are depression and anxiety symptoms.

The study set out to answer the following questions and associated hypotheses:

1. Is there a difference between maternal and paternal probable ASD/ PTSD in terms of prevalence and symptoms (clusters, severity, frequency)? We hypothesized that the ASD/ PTSD prevalence would be higher in mothers than fathers, that ASD/ PTSD symptoms would be higher in mothers, and that mothers would suffer more from arousal, avoidance, and intrusions.
2. Is ASD a predictor of PTSD after childbirth? We hypothesized that ASD would be a significant predictor of PTSD in mothers and fathers after controlling for confounders such as anxiety/depression, previous traumatic birth and the perinatal loss of a previous child (e.g., miscarriage, stillbirth). We also hypothesized that the predictive aspect of ASD would be stronger for mothers than fathers.
3. Is there a difference between maternal and paternal ASD, PTSD according to the type of birth: vaginal birth (VB), planned cesarean section (PCS) vs. unplanned operative birth (UOB) (e.g., forceps, ventouse and emergency cesarean section)? Our hypothesis was that ASD and PTSD prevalence would be higher for those who had an UOB compared to those who had a VB or a PCS, for both fathers and mothers, respectively.

## Materials and Methods

### Design

This study is part of a larger ongoing prospective population-based cohort (Lausanne Well-being Cohort), where data is collected during the third trimester of pregnancy up to 6 months post-partum.

### Participant Recruitment

Women in their third trimester of pregnancy and their partner were recruited at the Department of Obstetrics and Gynecology at a Swiss University Hospital. This study concerns those recruited between January 2013 to February 2019.

This study used a convenience sampling method. Inclusion criteria were: pregnant women in the second or third trimester and their partner (both ≥ 18 years of age) intending to attend the birth. Participants were excluded if: they had an insufficient knowledge of French to complete the questionnaires or if they had an acute psychosis or a suicidal risk. Following childbirth, parents whose child had serious complications, parents who had a stillborn child or experienced the death of their child shortly after birth, or partners who had not attended the birth were excluded. Partners' presence at the birth was indicated by their or their partner's response to a question about who was present at the birth (note that this question was only introduced into the cohort study later) or completed the Peritraumatic Dissociation Questionnaire [French Version; PDQ-FR; (57)], completed at 1 week. The rationale here was that the partner would not be able to complete this questionnaire if they had not been present at the birth.

A power analysis was conducted on the basis of a power of 0.80 and a 0.05 alpha to detect differences in means of ASD and PTSD symptoms, with effect sizes drawn from previous studies using the same instruments for ASD and PTSD (30, 36). This analysis estimated a sample size of 143 participants in each group, then 15% was added to account for attrition. The total expected sample size was therefore 330: 165 mothers and 165 fathers.

### Data Collection

Fathers and mothers separately completed online questionnaires at all time points. This prospective study used self-report questionnaires from three time points of the Cohort: during the third trimester of pregnancy (T1), 1 week post-partum (T2), and 1 month post-partum (T3). Hospital record data (obstetric and neonatal data) was extracted shortly after birth.

### Instruments

#### ASDS-F

ASD symptoms were assessed at 1 week post-partum using the 14-item validated instrument Acute Stress Disorder Scale (ASDS), (58, 59). Participants rated symptom items on a Likert scale from 1 (not at all) to 5 (a lot). Individual item scores are summed up and probable ASD is considered when endorsing at least 9 symptoms (3). For this study, the Cronbach α = 0.83.

#### PDS-F

PTSD was assessed 1 month post-partum using the validated 17 item self-report questionnaire Post-traumatic Diagnostic Scale-French version (PDS-F) (60). Participants were asked to complete the questionnaire in relation to a previous traumatic event that they were asked to identify at T1 and in relation to their childbirth at T3. Items are rated on a Likert scale from 0 (not at all) to 3 (more than five times a week); if an item has a response of 1 or higher, the symptom is considered as present. If responses corresponded to at least one re-experiencing symptom, three avoidance symptoms, and two arousal symptoms, participants were considered probable PTSD (61). Items were summed to create a total score, reflecting the severity of PTSD symptoms. For this study, the Cronbach α = 0.88.

#### HADS-F

The 14-item Hospital Anxiety and Depression Scale (HADS) was completed as a validated instrument of general psychological distress of parents at all time points: Seven items assess anxiety and seven items assess depression symptoms (62, 63). Items are rated on a Likert scale from 0 (no, not at all) to 3 (yes, definitely). Total scores were calculated by summing all items, the items of the two subscales were summed to create separate anxiety and depression scores. A higher score indicates greater severity of psychological distress, anxiety or depression. The cutoff used was as follows: above 14 was considered to have probable anxiety/depression (62). For this study, the Cronbach α for the HADS total and subscales was acceptable [HADS_anxiety:_ α = 0.72 (T1)–α = 0.79 (T2); HADS_depression:_ α = 0.66 (T1)–α = 0.79 (T2); HADS_total_ α = 0.76 (T1)–α = 0.86 (T2)].

### Data Analyses

Analyses were carried out using STATA version 13. Prevalence for ASD and PTSD diagnosis was first established for the entire sample according to gender, then by gender and birth mode. Regarding hypothesis 2 and 3, we conducted regression models, where potential confounders identified from the literature that showed a significant correlation with the dependent variable were included.

Test of proportions or chi-squared tests were used to investigate gender differences in the prevalence of ASD and PTSD diagnosis, and if there was a difference between genders in each birth group. To further investigate this question, we conducted a logistic regression for mothers and fathers to see if there was a relation between the mode of birth and ASD prevalence. Symptoms of ASD and PTSD were compared according to gender, using a Mann Whitney U-test, independently of the birth mode. Following hypothesis 1, unilateral tests were performed.

Second, to determine if PTSD diagnosis was associated with ASD diagnosis after birth according to gender, a logistic regression was conducted, with PTSD at 1 month post-partum as the dependent variable. We also conducted a linear regression to determine if PTSD severity was associated with ASD severity. According to the model of the etiology of PTSD-CB, we tested the correlations between cited confounders and ASD and PTSD. In both models, ASD and gender were included as independent variables with anxiety/depression at 1 week post-partum and previous perinatal loss as covariates if significantly correlated.

### Ethical Considerations

This study was approved by the ethics committee of the Canton Vaud, Switzerland (approval number: 480/2012). Written informed consent was obtained from participants. They all had the possibility to ask questions or to withdraw from the study at any time and without explanation. All participant data was coded.

## Results

### Participant Characteristics

The final sample was composed of 647 participants (419 birthing mothers, 228 fathers). At T1, 385 mothers and 209 fathers completed the questionnaires, 81.8% of mothers and 73.2% of fathers returned the questionnaires at T2, and 76.6% of mothers and 64.8% of fathers at T3. In total, 178 couples are represented in the study.

During pregnancy, mean maternal age was 32.8 ± 4.5, range 18 to 45 years and mean paternal age was 34.2 ± 5.25, range 18 to 53 years. Most of the participants (73%) were cohabiting or married, and 65% had an education level beyond high school (see Table 1). 63% of participants were expecting their first child. 56% of mothers had a VB and 27% had an UOB, of which 17% were unplanned C-sections.

**Table 1:** Sociodemographic characteristics at T1 for mothers, fathers, obstetric and neonatal variables.

	**Frequency (%)**	**Mean (SD)**
**Maternal sociodemographic variables**		
Age (years; mean, SD)		32.8 (4.5)
Nationality (*n*, %)		
Swiss	13 (3.6%)	
Other European	224 (61.2%)	
Non-European	129 (35.3%)	
Migration status (*n*, %)		
Migrant	89 (37.6%)	
Non-migrant	148 (62.5%)	
Marital status (*n*, %)		
Single	96 (22.9%)	
Co-habiting/Married	312 (74.6%)	
Divorced	6 (1.4%)	
Education (n, %)		
Middle school	17 (4.2%)	
Secondary/high school	28 (7%)	
Apprenticeship	80 (19.9%)	
University	256 (63.5%)	
Largo score (mean, SD)^†^		7.68 (1.22)
**Obstetric variables**		
Parity (mean, SD)		0.49 (0.69)
Gestity (mean, SD)		1.80 (0.96)
Arterial cord blood pH (mean, SD)		7.24 (0.08)
Venous cord blood pH (mean, SD)		7.31 (0.07)
Delivery mode		
Vaginal birth	285 (56.2%)	
Planned cesarean section (PCS)	82 (16.2%)	
Unplanned operative birth (UOB)	140 (27.6%)	
Ventouse, forceps	54 (10.6%)	
Unplanned C-section	86 (17%)	
**Paternal sociodemographic variables**		
Age (years; mean, SD)		34.25 (5.25)
Nationality (*n*, %)		
Swiss	8 (3.8%)	
Other European	129 (61.4%)	
Non-European	73 (34.8%)	
Migration status (n, %)		
Migrant	50 (37%)	
Non-migrant	85 (63%)	
Marital status (*n*, %)		
Single	56 (24.7%)	
Co-habiting/Married	164 (72.3%)	
Divorced	6 (2.6%)	
Education (*n*, %)		
Middle school	9 (4.1%)	
Secondary/high school	6 (2.8%)	
Apprenticeship	40 (18.4%)	
University	152 (69.7%)	
Largo score (mean, SD)^†^		7.68 (0.094)

† *Largo score: parental socioeconomic status was obtained by adding the education level of the mother and the professional activity of the father (64)*.

There were no sociodemographic differences between mothers and fathers, except in age (*z* = −3.01; *p* = 0.0026; *d* = 0.296), where mothers were younger than fathers. Participants who did not complete the ASDS at T2 had the same characteristics as those who responded at T1 (data not shown). For PTSD symptoms at T3, the participants who did not answer the questionnaire did not differ from T1, except for the proportion of individuals of European origin (*z* = −2.31; *p* = 0.0211, *h* = 0.203) and those having an education level below secondary/high school (primary school: *z* = 1.97; *p* = 0.049; *h* = 0.651, secondary school: *z* = 2.59; *p* = 0.0097; *h* = 0.912).

#### Mental Health of Participants During the Third Trimester of Pregnancy

Prevalence rates for probable PTSD diagnosis in the third trimester was 24.3% for mothers and 20.3% for fathers. The mean score for maternal PTSD symptoms was 7.37 ± 8.01 and for paternal PTSD symptoms was 6.54 ± 7.08. Neither rates nor means differed significantly (*p* = ns). 1/3 of mothers and fathers had experienced a previous traumatic event (e.g., car accident, sexual abuse). More mothers (16.2%) than fathers (9%) had experienced a previous traumatic childbirth (*z* = 2.29; *p* = 0.0220; *h* = 0.213). 23.5% of mothers and 18.6% of fathers had experienced a previous perinatal loss but this difference was non-significant (*p* = ns).

#### Anxiety and Depression Symptoms of Parents During the Study

Mothers had a higher prevalence of psychological distress and higher means of anxiety and depression symptoms than fathers, at all time points (see Table 2).

**Table 2:** Parental psychological distress during the study.

	**T1**	**T2**	**T3**
**General psychological distress (%)**			
Mothers	20.2%^*^	30.86%^*^	24.12%^*^
Fathers	10.9%^*^	12.09%^*^	12.36%^*^
All	16.82%	24.11%	19.83%
**HADS severity [mean; (SD)]**			
Mothers	10.91 (5.20)^*^	11.66 (6.82)^*^	11.04 (5.94)^*^
Fathers	8.70 (4.56)^*^	7.86 (6.20)^*^	7.8 (5.21)^*^
All	10.10 (5.07)	10.29 (6.82)	9.86 (5.88)
**Anxiety (mean; SD)**			
Mothers	6.88 (3.18)^*^	6.50 (3.77)^*^	6.40 (3.56)^*^
Fathers	6.07 (3.09)^*^	5.01 (3.18)^*^	4.64 (2.89)^*^
All	6.60 (3.17)	6.02 (3.65)	5.83 (3.46)
**Depression (mean; SD)**			
Mothers	3.35 (2.57)^*^	4.32 (4.13)^*^	3.71 (3.21)^*^
Fathers	3.71 (2.92)^*^	4.56 (3.57)^*^	4.44 (3.29)^*^
All	3.55 (2.77)	4.46 (3.80)	4.15 (3.27)

* *p < 0.050*.

### Prevalence and Symptoms Severity of ASD Symptoms Following Childbirth

At 1 week post-partum (T2), 8.9% of mothers and 4.4% of fathers showed symptoms of probable ASD, regardless of the delivery mode. The difference in prevalence found between mothers and fathers regardless of the delivery mode was non-significant, and informing hypothesis 1. 5% of mothers with a VB reported ASD symptoms compared to 10.7 and 18.7% of those who respectively had a PCS and an UOB. Fifty percent of fathers attending a VB had probable ASD compared to 43.7 and 55% of those who, respectively attended a PCS and an UOB. Regarding the delivery mode, the difference was found to be non-significant for mothers and fathers (*p* = ns). Thus informing hypothesis 3. However, in the logistic regression [$\chi_{\left(4,\text{N}=128\right)}^{2}$ = 12.9; *p* = 0.0118] where we included significant psychological and sociodemographic covariates (e.g., age, anxiety/depression, previous perinatal loss) (see Table 3), mothers who had an UOB had a higher risk (OR = 3.1) to develop ASD (see Table 4). For fathers, the logistic regression was not possible, as no fathers who attended a PCS had probable ASD.

**Table 3:** Bivariate Pearson correlations with probable ASD and PTSD diagnosis and with PTSD severity at 1 month post-partum.

	**ASD probable diagnosis (mothers; fathers)**	**PTSD probable diagnosis (mothers; fathers; both parents^a^)**	**PTSD** **severity**	
Gender			−0.17^*^^a^	−0.24^*^^b^
Age	0.00; 0.04	0.00; −0.00	−0.02^a^	−0.03^b^
Socio-economic status (Largo)	0.08: 0.05	0.04; 0.23^*^	0.07^a^	0.08^b^
Anxiety (pregnancy 3rd trimester)	0.14; 0.12			
Depression (pregnancy 3rd trimester)	0.17^*^; 0.12			
Anxiety (at 1 week post-partum)		0.39^*^; 0.32^*^	0.40^*^^a^	0.53^*^^b^
Depression (at 1 week post-partum)		0.43^*^; 0.44^*^	0.46^*^^a^	0.56^*^^b^
ASD probable diagnosis			0.43^*^^a^	
ASD severity				0.73^*^^b^
Previous traumatic birth	0.04; 0.04	0.03; −0.09	0.02	0.04^b^
Previous perinatal loss	0.01; −0.16	0.15^*^; 0.08	0.14^*^^a^	0.06^b^

* *p < 0.050*.

a *Correlations calculated regardless of the gender as it was an independent variable in the logistic regression of PTSD at 1 month*.

b *Correlations calculated regardless of the gender as it was an independent variable in the regression of PTSD (continuous scores)*.

**Table 4:** Logistic regression of ASD at 1 week for mothers according to type of birth.

**ASD at 1 week**	**Odd ratio**	***p-value***	**95% confidence interval**	
Depression in 3rd trimester	1.28	0.025	1.03	1.59
Mode of birth:				
Planned C-section	2.44	0.313	0.43	13.81
Unplanned operative birth	7.02	0.012	1.55	31.88

For ASD symptoms, the mean severity score for mothers was 21.58 (SD = 7.21) and 18.83 (SD = 4.42) for fathers. The difference in the ASD symptom severity was significant (*z* = 2.76; *p* = 0.0058; *d* = 0.433) and in line with hypothesis 1. The four symptom clusters of ASD were also higher for mothers than fathers, as hypothesized (see Table 5).

**Table 5:** Differences in ASD symptom severity according to gender (*T*-test).

	**Mothers**	**Fathers**	***z***	***p***	***d***
	(*n* = 306)	(*n* = 150)			
Arousal symptoms [mean (SD)]	8.95 (3.90)	7.88 (2.73)	2.40	0.0165	0.302
	(*n* = 158)	(*n* = 91)			
Avoidance symptoms [mean (SD)]	3.94 (1.52)	3.61 (1.10)	1.69	0.0451	0.236
	(*n* = 308)	(*n* = 151)			
Negative emotions symptoms [mean (SD)]	2.81 (1.33)	2.43 (0.92)	3.37	0.0008	0.307
Reexperiencing symptoms [mean (SD)]	5.62 (2.60)	4.92 (1.44)	2.27	0.0232	0.309

### Prevalence and Symptoms Severity of PTSD Symptoms Following Childbirth

At 1 month post-partum (T3), 20.7% of mothers and 7.2% of fathers had probable PTSD, regardless of delivery mode. This difference was significant [$\chi_{\left(1,\text{N}=424\right)}^{2}$ = 12.51; *p* < 0.0001; *h* = 0.391] and in accordance with hypothesis 1. Some mothers (18.8%) with a VB presented PTSD symptoms compared to 21.6 and 27.3% of those who respectively had a PCS and an UOB. Fathers attending a VB were 6.6% to report PTSD symptoms compared to 9.5 and 10% of those who, respectively attended a PCS and an UOB. However, none of these differences was significant (both *p*=ns). In the logistic regression [$\chi_{\left(5,\text{N}=108\right)}^{2}$ = 31.43; *p* < 0.00], the whole model was significant. Anxiety and depression symptoms showed a significant influence on probable PTSD at 1 month post-partum but not on mode of birth (see Table 6). For fathers, this analysis could not be not performed due to small sample size (men who attended a PCS and had a probable PTSD = 2).

**Table 6:** Logistic regression of PTSD at 1 month for mothers according to type of birth.

**PTSD at 1 week**	**Odd ratio**	***p-*value**	**95% confidence interval**	
Anxiety at 1 week post-partum	1.22	0.005	1.06	1.4
Depression at 1 week post-partum	1.16	0.03	1.01	1.32
Previous perinatal loss	2.61	0.073	0.92	7.43
Mode of birth:
Planned C-section	1.54	0.516	0.42	5.72
Unplanned operative birth	2.71	0.083	0.88	8.4

For PTSD symptoms, the mean severity score for mothers was 6.78 (SD = 6.94) and 3.54 (SD = 4.49) for fathers. This difference was significant (*z* = 5.46; *p* < 0.0001; *d* = 0.517) and in accordance with hypothesis 1. The three symptom cluster scores of PTSD were also higher for mothers than fathers, as predicted in hypothesis 1 (see Table 7).

**Table 7:** Differences in PTSD symptom severity according to gender (*T*-test).

	**Mothers**	**Fathers**	***z***	***p***	***d***
	(*n* = 286)	(*n* = 141)			
Arousal symptoms [mean (SD)]	3.33 (3.23)	1.85 (2.21)	4.64	0.0165	0.502
	(*n* = 287)	(*n* = 139)			
Avoidance symptoms [mean (SD)]	2.08 (2.90)	1.19 (2.16)	3.71	0.0002	0.332
	(*n* = 288)	(*n* = 141)			
Reexperiencing symptoms [mean (SD)]	1.38 (2.26)	0.54 (1.41)	4.99	<0.001	0.415

### ASD as a Predictor of PTSD After Childbirth

ASD was a predictor of PTSD: 81.3% of parents with probable ASD had probable PTSD after 1 month, whereas 84.9% of parents without probable ASD did not develop probable PTSD at 1 month post-partum (see Table 8). When participants had probable ASD, their risk to develop probable PTSD was 14 times than those without probable ASD, even after including gender, anxiety/depression at 1 week, and previous perinatal loss in the regression. The regression model was significant [$\chi_{\left(5,\text{N}=182\right)}^{2}$ = 65.91; *p* < 0.0001] and explained 33.9% of the variance of probable PTSD diagnosis (see Table 9).

**Table 8:** Proportions of mothers and fathers having PTSD regarding ASD status.

	**No ASD: *n* (%) mothers; fathers**	**ASD: *n* (%) mothers; fathers**
No PTSD (*N*; %)	101 (79.53%)^a^; 73 (93.59%)^b^	2 (15.38%)^a^; 1 (33.33%)^b^
PTSD (*N*; %)	26 (20.47%)^a^; 5 (6.41)^b^	11 (84.62%)^a^; 2 (66.67%)^b^

a *[$\chi_{\left(1,\text{N}=140\right)}^{2}$ = 24.95; p < 0.0001]*.

b *[$\chi_{\left(1,N=81\right)}^{2}$ = 13.28; p = 0.019]*.

**Table 9:** Logistic regression of PTSD at 1 month.

**PTSD at 1 month**	**Odd ratio**	***p-value***	**95% confidence interval**	
ASD	14.00	0.002	2.55	76.98
Gender	0.52	0.248	0.17	1.56
Anxiety at 1 week	1.08	0.249	0.94	1.25
Depression at 1 week	1.27	0.001	1.11	1.47
Previous perinatal loss	1.77	0.234	0.69	4.56

According to the regression model (Table 10), the ASD severity score was also a predictor of the PTSD severity score at 1 month post-partum. The model [$\chi_{\left(4,\text{N}=219\right]}^{2}$ = 72.83; *p* < 0.0001] explained 57% of the variance of PTSD symptoms severity score.

**Table 10:** Regression of PTSD at 1 month (continuous score).

**PTSD severity at 1 month**	**coefficient**	***p-value***	**95% confidence interval**	
ASD severity	0.55	<0.0001	0.43	0.67
Gender	−0.87	0.192	−2.19	0.44
Anxiety at 1 week	0.14	0.194	−0.07	0.36
Depression at 1 week	0.25	0.029	0.03	0.47
Previous perinatal loss	0.01	0.984	−1.34	1.37

To determine if probable ASD was a stronger predictor of probable PTSD for mothers than fathers, a logistic regression was conducted, where probable ASD and gender were combined. However, this was not possible, as the number of fathers with probable PTSD was insufficient. Thus, a linear regression was conducted, including an interaction between ASD symptoms and gender. No differences in the predictive aspect of ASD symptoms according to gender were found (Table 11). The model [$\chi_{\left(5,\text{N}=219\right)}^{2}$ = 58.02; *p* < 0.0001] explained 58% of the variance of PTSD.

**Table 11:** Regression of PTSD (continuous score) at 1 month with interaction of ASD and gender.

**PTSD at 1 month**	**Coefficient**	***p-*value**	**95% confidence interval**	
ASD severity	0.55	<0.0001	0.42	0.67
Gender: men	−1.58	0.533	−6.58	3.42
Gender and ASD combined	−0.36	0.772	−0.21	0.29
Anxiety at 1 week	0.14	0.207	−0.08	0.36
Depression at 1 week	0.25	0.029	0.03	0.47
Previous perinatal loss	0.02	0.977	−1.34	1.38

## Discussion

This prospective population-based cohort study firstly aimed to compare the prevalence of ASD and PTSD probable diagnosis and symptoms severity between mothers and fathers following childbirth, while considering other comorbid variables, such as anxiety, depression, obstetric variables, and previous perinatal loss. The second aim of this study was to determine if probable ASD was a predictor of probable PTSD after childbirth. We found that 8.9% of mothers and 4.4% of fathers presented symptoms of ASD at 1 week post-partum; 20.7% of mothers and 7.2% of fathers reported PTSD symptoms after 1 month, confirming hypothesized gender differences. Probable ASD was found to be a predictor of probable PTSD after childbirth, confirming hypothesis 2. Those findings are important because they underline the importance of early screening of ASD and its potential relation to future PTSD development.

It is worth noting that in the third trimester, probable PTSD rates were 20.3% for fathers and 24.3% for mothers. These rates correspond to high risk samples (10). In our study, this can perhaps be explained by a previous traumatic birth (13.7%) or a previous perinatal loss (21.8%) experienced by the participants, in line with the theoretical framework highlighting previous traumatic birth or problems associated with previous pregnancy as risk factors for PTSD (54).

Unlike prevalence rates of PTSD-CB, prevalence of general psychological distress in the third trimester (mothers: 20.2%; fathers: 10.9%) is more in line with community samples in the literature (8, 31). In our study, only depression symptoms showed as a significant confounder in the prediction of PTSD. However, this may be due to the relatively low level of anxiety in the sample in comparison to the high scores of PTSD symptoms.

The maternal prevalence rate of probable ASD diagnosis, regardless of the delivery mode, was in line with an other study in the same context (12), but was lower than rates found in the NICU (35, 38, 48, 65). The probable PTSD rates in the third trimester indicate that our sample corresponds more to a high-risk sample.

The prevalence of probable PTSD diagnosis regardless of the delivery mode in our study of 20.7% for mothers at 1 month post-partum is in accordance with a systematic review reporting a rate of 18.5% for high-risk samples (11). The suggestion that we have a high-risk sample from the third trimester data is born out post-partum, for mothers at least. For fathers, given the scarcity of studies, our prevalence rate of probable PTSD of 7.2% is within the current estimates: 0 to 5% (27, 28, 31), with a maximum of 12% depending on the sampling method (30).

As hypothesized, in our study, gender differences were found in ASD and PTSD (prevalence, symptoms and clusters) between mothers and fathers. Mothers had a significantly higher rate of probable ASD diagnosis and symptom severity than fathers. These gender differences in severity score add to the evidence of gender differences in NICU parents (38, 48). Gender differences in probable ASD diagnosis are less consistent. Our results correspond to some studies in NICU parents (35, 38), though contrasts with another, where differences were found (48). Whether differences in evidence are due to methodological differences or other factors, such as reasons for NICU admission, are as yet unclear and should be further investigated.

Mothers also showed a higher probable PTSD diagnosis rate and symptom severity than fathers. Such gender differences in probable diagnosis rates have also been seen in other studies (37), though these differences were not always significant (33, 35). Gender differences in symptom severity scores are also in line with previous studies (27, 30) Gender differences in ASD and PTSD could possibly be explained by “living” (direct exposure) being more predictive of ASD than “witnessing” (indirect exposure) a traumatic event, or that fathers put their spouse's needs first, resulting in the tendency to underreport their symptoms (27). Symptom severity was higher for mothers following birth across all ASD and PTSD symptom clusters. In the NICU, studies also found a significant difference for re-experiencing symptoms and arousal (32, 33, 37). Mothers may report more arousal symptoms, as they include sleep disturbance, a common symptom in the post-partum period (16).

Contrary to previous studies (1, 54, 66), we found no evidence that OB (PCS or UOB) is a risk factor for either ASD or PTSD symptoms in mothers. There was no significant difference in probable ASD diagnosis rates for mothers or fathers regarding the delivery mode. However, in the logistic regression models, the risk to develop ASD was 7.02 times higher in mothers following UOB. Therefore, our hypothesis that there would be differences between probable parental PTSD and ASD by birth mode was not supported. Results may be explained by the fact that, according to Beck (67), “what is found traumatic lies in the eye of the beholder” (p.2). This underlines the importance of the subjective birth experience, as found in some studies (10, 54, 68, 69). According to Garthus-Niegel et al. (68), even if the objective birth experience can lead to PTSD symptoms, the subjective birth experience is much more important. The lack of difference across birth modes for fathers may also indicate that birth mode may not carry the same risk for ASD and PTSD diagnosis in fathers. Indeed, a previous study found that childbirth is a traumatic event for men, regardless of delivery mode (70). Therefore, future studies should further investigate risk factors for fathers, as mothers and fathers may not share the same.

Although many mothers and fathers with ASD symptoms at T2 did not go on to develop PTSD symptoms at T3, ASD was found to be a predictor of PTSD at 1 month post-partum across probable PTSD diagnosis and symptom severity scores. This finding is supported by several studies in different contexts, like the NICU or PICU (40, 42). However, other studies found that ASD was not specific enough to predict PTSD (43–45). We found that 84.9% of parents without probable ASD did not develop probable PTSD post-partum, whereas 81.3% of parents with probable ASD had probable PTSD. Similar rates were found in the PICU (42). Identifying those with ASD and intervening with a low-level intervention may go a long way to alleviate distressing symptoms in the immediate aftermath post-partum, but also prevent PTSD from developing in a third of parents. Our results suggest that probable ASD is a greater predictor in mothers, adding nuance to this suggestion. Indeed, 84.6% of mothers with probable ASD developed probable PTSD, compared to 66.7% of fathers. Thus, interventions following ASD screening may be more effective in reducing the risk of full PTSD for mothers. The lower rates of fathers with probable ASD going on to have probable PTSD again, reiterates the idea that perhaps the risk factors in PTSD development for fathers are different from those for mothers; through identification of these factors, more targeted interventions may be possible for fathers.

### Strengths and Limitations

A major strength of our prospective population-based study is that we were able to compare self-reported *parental* ASD and PTSD symptoms and probable diagnosis after childbirth. Furthermore, comorbid variables, such as mode of birth, previous perinatal loss, as well as anxiety and depression symptoms, were measured at all time points and included in all regressions assessing ASD as a predictor of PTSD. Additional strengths of our study were the large sample size including mothers and fathers and the relatively high response rates.

An important limitation of the study was the convenience sample (homogeneity): all participants were recruited in the same university hospital and had to speak French, thus limiting the generalizability of the findings. The measurement of anxiety/depression, ASD and PTSD with self-report questionnaires, rather than by clinical interviews, may have overestimated the prevalence of psychological distress, as well as probable ASD and PTSD (11, 71). In addition, it should be noted that the assessment of PTSD in our study was based on DSM-IV, as no validated self-report measure for PTSD according to DSM-5 was available at the beginning of the study. Finally, it is possible that “false positives” were identified when conducting routine assessment or screening in the post-partum context, as some factors commonly associated with having a newborn (e.g., increased vigilance) can overestimate rates of PTSD following childbirth (72).

### Clinical Implications and Suggestions for Future Research

The relatively high proportion of mothers and fathers experiencing PTSD symptoms as well as probable diagnosis confirms that childbirth can be a traumatic event for *both* parents. In addition, the perinatal period can be distressing for *both* parents, with approximately one fourth of the parents presenting with anxiety and depression symptoms. Therefore, it is important to also care for fathers. Healthcare professionals should strive to better include fathers in the antenatal and post-natal checkups (73).

The Odds ratio of probable ASD diagnosis was higher for mothers following UOB, compared to mothers following VB or PCS. This suggests that birth mode may be influencing the mother's experience of her birth, resulting in trauma symptoms in the immediate aftermath. However, this was not the case for probable PTSD diagnosis. While birth mode may not influence directly PTSD, it may show an influence through ASD. Good preparation of the mother, in particular, for what happens during an operative birth, may function as a prevention method for both ASD and subsequent PTSD development.

Early screening of ASD symptoms would appear to be a good prevention method to detect parents at risk of developing PTSD, particularly for mothers. It is currently recommended to assess anxiety/depression symptoms and psychological well-being of parents as soon as possible during the pregnancy and after birth (74, 75). This should also include ASD. Therefore, all healthcare professionals, including nurses and midwives on post-partum wards, should be trained to recognize ASD. They are best placed to observe and care for mothers during their hospital stay, as well as interact with fathers. This would allow the timely referral of parents to mental health specialists for those who are in need.

More research is required to fully understand the most important risk factors for fathers' development of PTSD-CB. Obstetric variables for fathers should be measured in future studies. Our evidence suggests that partners may have different risk factors, or risk factors may have a different relative importance, compared to mothers. Finally, future studies should assess PTSD with a self-report questionnaire based on DSM-5 criteria or make use of clinical diagnostic tools.

## Conclusion

This study shows that probable ASD diagnosis after childbirth is present in *both* mothers and fathers. Prevalence of probable PTSD diagnosis after 1 month was one fifth for mothers and 7% for fathers. Caution should be taken when interpreting those prevalence rates, as the sample in our study corresponds to a high-risk sample. Mothers presented higher scores of ASD and PTSD symptoms than fathers. Probable ASD diagnosis was found to be a predictor of probable PTSD diagnosis 1 month after childbirth. Early screening of ASD symptoms may help identify parents at risk of developing PTSD.

## Data Availability Statement

The raw data supporting the conclusions of this article will be made available by the authors, without undue reservation.

## Ethics Statement

The studies involving human participants were reviewed and approved by the ethics committee of the Canton Vaud, Switzerland. The patients/participants provided their written informed consent to participate in this study.

## Author Contributions

AH conceived of the study and its design, and coordinated the data collection, participated in the data analysis, and critically reviewed the manuscript. SS participated in the coordination of the study and critically reviewed the manuscript. ES conducted part of the data collection and data analysis under supervision of SS and AH. All authors co-wrote parts of the manuscript. All authors read and approved the final manuscript.

## Conflict of Interest

The authors declare that the research was conducted in the absence of any commercial or financial relationships that could be construed as a potential conflict of interest.

## Publisher's Note

All claims expressed in this article are solely those of the authors and do not necessarily represent those of their affiliated organizations, or those of the publisher, the editors and the reviewers. Any product that may be evaluated in this article, or claim that may be made by its manufacturer, is not guaranteed or endorsed by the publisher.
