# Corrigendum: Acute and Post-traumatic Stress Disorder Symptoms in Mothers and Fathers Following Childbirth: A Prospective Cohort Study

**DOI:** 10.3389/fpsyt.2022.790170

**Published:** 2022-02-11

**Authors:** Elisabeth Schobinger, Suzannah Stuijfzand, Antje Horsch

**Affiliations:** ^1^Institute of Higher Education and Research in Healthcare in French, University of Lausanne, Lausanne, Switzerland; ^2^Department Woman-Mother-Child, Lausanne University Hospital, Lausanne, Switzerland

**Keywords:** acute stress (disorder), PTSD-post-traumatic stress disorder, parents, mothers, fathers, nurses and midwives, childbirth

In the original article, there was an error. The cut-off for the Acute Stress Disorder Scale-French version (ASDS-F) was miscalculated, thus resulting in wrong prevalence rates of acute stress disorder (ASD). Given that these prevalence rates were then used for further analyses, corrections in a variety of places in the paper had to be made.

A correction has been made to **Abstract, Results**:

“At T2, 8.9% of mothers and 4.4% of fathers presented symptoms of acute stress disorder.”

A correction has been made to **Material and Methods, Instruments**, **ASDS-F**:

“Individual item scores are summed up and probable ASD is considered when endorsing at least 9 symptoms (3).”

A correction has been made to **Results, Prevalence and Symptoms Severity of ASD Symptoms Following Childbirth**, paragraph one, paragraph two:

“At 1 week post-partum (T2), 8.9% of mothers and 4.4% of fathers showed symptoms of probable ASD, regardless of the delivery mode. The difference in prevalence found between mothers and fathers regardless of the delivery mode was non-significant, and informing hypothesis 1. 5% of mothers with a VB reported ASD symptoms compared to 10.7 and 18.7% of those who respectively had a PCS and an UOB. […] For fathers, the logistic regression was not possible, as no fathers who attended a PCS had probable ASD.”

“The four symptom clusters of ASD were also higher for mothers than fathers, as hypothesized (see Table 5).”

A correction has been made to **Results, ASD as a predictor of PTSD after Childbirth**, paragraph one:

“ASD was a predictor of PTSD: 81.3% of parents with probable ASD had probable PTSD after 1 month, whereas 84.9% of parents without probable ASD did not develop probable PTSD at 1 month post-partum (see **Table 8**). When participants had probable ASD, their risk to develop probable PTSD was 14 times than those without probable ASD, even after including gender, anxiety/depression at 1 week, and previous perinatal loss in the regression. The regression model was significant [$\chi_{\left(5,\text{N}=182\right)}^{2}$ = 65.91; *p* < 0.0001] and explained 33.9% of the variance of probable PTSD diagnosis (see **Table 9**).”

A correction has been made to **Discussion**, paragraph one:

“We found that 8.9% of mothers and 4.4% of fathers presented symptoms of ASD at 1 week post-partum; 20.7% of mothers and 7.2% of fathers reported PTSD symptoms after 1 month, confirming hypothesized gender differences.”

A correction has been made to **Discussion**, paragraph three, paragraph four:

“Unlike prevalence rates of PTSD-CB, prevalence of general psychological distress in the third trimester (mothers: 20.2%; fathers: 10.9%) is more in line with community samples in the literature (8, 31). In our study, only depression symptoms showed as a significant confounder in the prediction of PTSD. However, this may be due to the relatively low level of anxiety in the sample in comparison to the high scores of PTSD symptoms.”

“The maternal prevalence rate of probable ASD diagnosis, regardless of the delivery mode, was in line with an other study in the same context (12), but was lower than rates found in the NICU (35, 38, 48, 65). The probable PTSD rates in the third trimester indicate that our sample corresponds more to a high-risk sample.”

A correction has been made to **Discussion**, paragraph five, paragraph six:

“For fathers, given the scarcity of studies, our prevalence rate of probable PTSD of 7.2% is within the current estimates: 0 to 5% (27, 28, 31), with a maximum of 12% depending on the sampling method (30).”

“Our results correspond to some studies in NICU parents (35, 38), though contrasts with another, where differences were found (48).”

A correction has been made to **Discussion**, paragraph eight, paragraph nine:

“However, in the logistic regression models, the risk to develop ASD was 7.02 times higher in mothers following UOB.”

“We found that 84.9% of parents without probable ASD did not develop probable PTSD post-partum, whereas 81.3% of parents with probable ASD had probable PTSD. […] Indeed, 84.6% of mothers with probable ASD developed probable PTSD, compared to 66.7% of fathers.”

A correction has been made to **Discussion, Clinical Implications and Suggestions for Future Research**, paragraph one:

“The relatively high proportion of mothers and fathers experiencing PTSD symptoms as well as probable diagnosis confirms that childbirth can be a traumatic event for *both* parents.”

A correction was has been made to **Conclusion**:

“This study shows that probable ASD diagnosis after childbirth is present in *both* mothers and fathers. Prevalence of probable PTSD diagnosis after 1 month was one fifth for mothers and 7% for fathers.”

In the original article, there was an error. Table citations were incorrect in some places.

A correction has been made to **Results, Prevalence and Symptoms Severity of PTSD Symptoms following Childbirth**, paragraph one, paragraph two:

“Anxiety and depression symptoms showed a significant influence on probable PTSD at 1 month post-partum but not on mode of birth (see Table 6).”

“The three symptom cluster scores of PTSD were also higher for mothers than fathers, as predicted in hypothesis 1 (see Table 7).”

A correction was made in **Results, ASD as a Predictor of PTSD After Childbirth**, paragraph two, paragraph three:

“According to the regression model (Table 10), the ASD severity score was also a predictor of the PTSD severity score at 1 month post-partum.”

“No differences in the predictive aspect of ASD symptoms according to gender were found (Table 11).”

In the original article, there was a mistake in Table 3 as published. The wrong correlation coefficients were reported. The corrected Table 3 appears at the end of this article.

**Table 3:** Bivariate Pearson correlations with probable ASD and PTSD diagnosis and with PTSD severity at 1 month post-partum.

	**ASD probable diagnosis (mothers; fathers)**	**PTSD probable diagnosis (mothers; fathers; both parents^**a**^)**	**PTSD** **severity**	
Gender			−0.17^*^^a^	−0.24^*^^b^
Age	0.00; 0.04	0.00; −0.00	−0.02^a^	−0.03^b^
Socio-economic status (Largo)	0.08: 0.05	0.04; 0.23^*^	0.07^a^	0.08^b^
Anxiety (pregnancy 3rd trimester)	0.14; 0.12			
Depression (pregnancy 3rd trimester)	0.17^*^; 0.12			
Anxiety (at 1 week post-partum)		0.39^*^; 0.32^*^	0.40^*^^a^	0.53^*^^b^
Depression (at 1 week post-partum)		0.43^*^; 0.44^*^	0.46^*^^a^	0.56^*^^b^
ASD probable diagnosis			0.43^*^^a^	
ASD severity				0.73^*^^b^
Previous traumatic birth	0.04; 0.04	0.03; −0.09	0.02	0.04^b^
Previous perinatal loss	0.01; −0.16	0.15^*^; 0.08	0.14^*^^a^	0.06^b^

* *p < 0.050*.

a *Correlations calculated regardless of the gender as it was an independent variable in the logistic regression of PTSD at 1 month*.

b *Correlations calculated regardless of the gender as it was an independent variable in the regression of PTSD (continuous scores)*.

In the original article, there was a mistake in Table 4 as published. There was an error in the results due to the misinterpretation of ASDS cut-off. The corrected Table 4 appears at the end of this article.

**Table 4:** Logistic regression of ASD at 1 week for mothers according to type of birth.

**ASD at 1 week**	**Odd ratio**	***p-value***	**95% confidence interval**	
Depression in 3rd trimester	1.28	0.025	1.03	1.59
Mode of birth:				
Planned C-section	2.44	0.313	0.43	13.81
Unplanned operative birth	7.02	0.012	1.55	31.88

In the original article, there was a mistake in **Table 5** as published. Due to the misinterpretation of the ASD prevalence, these analyses were not possible. This table therefore does not exist anymore.

In the original article, there was a mistake in the numbering of **Table 6** as published. The corrected table numbering appears below.

“Table 5 Differences in ASD symptom severity according to gender (*T*-test).”

In the original article, there was a mistake in the numbering of **Table 7** as published. The corrected table numbering appears below.

“Table 6 Logistic regression of PTSD at 1 month for mothers according to type of birth.”

In the original article, there was a mistake in the numbering of Table 8 as published. The corrected table numbering appears below.

**Table 8:** Proportions of mothers and fathers having PTSD regarding ASD status.

	**No ASD: *n* (%) mothers; fathers**	**ASD: *n* (%) mothers; fathers**
No PTSD (*N*; %)	101 (79.53%)^a^; 73 (93.59%)^b^	2 (15.38%)^a^; 1 (33.33%)^b^
PTSD (*N*; %)	26 (20.47%)^a^; 5 (6.41)^b^	11 (84.62%)^a^; 2 (66.67%)^b^

a *[$\chi_{\left(1,\text{N}=140\right)}^{2}$ = 24.95; p < 0.0001]*.

b *[$\chi_{\left(1,N=81\right)}^{2}$ = 13.28; p = 0.019]*.

“Table 7 Differences in PTSD symptom severity according to gender (*T*-test).”

In the original article, there was a mistake in Table 9 as published. There was an error in the results due to the misinterpretation of the ASDS cut-off, and the table number was incorrect. The corrected table and table numbering appears at the end of this article.

**Table 9:** Logistic regression of PTSD at 1 month.

**PTSD at 1 month**	**Odd ratio**	***p-value***	**95% confidence interval**	
ASD	14.00	0.002	2.55	76.98
Gender	0.52	0.248	0.17	1.56
Anxiety at 1 week	1.08	0.249	0.94	1.25
Depression at 1 week	1.27	0.001	1.11	1.47
Previous perinatal loss	1.77	0.234	0.69	4.56

In the original article, there was a mistake in **Table 10** as published. There was an error in the results due to the misinterpretation of the ASDS cut-off, and the table number was incorrect. The corrected table and table numbering appears at the end of this article.

In the original article, there was a mistake in the numbering of **Table 11** as published. The corrected table numbering appears below.

“Table 10 Regression of PTSD at 1 month (continuous score).”

In the original article, there was a mistake in the numbering of **Table 12** as published. The corrected table numbering appears below.

“Table 11 Regression of PTSD (continuous score) at 1 month with interaction of ASD and gender.”

The authors apologize for these errors and confirm that this does not change the scientific conclusions of the article in any way. The original article has been updated.

## Publisher's Note

All claims expressed in this article are solely those of the authors and do not necessarily represent those of their affiliated organizations, or those of the publisher, the editors and the reviewers. Any product that may be evaluated in this article, or claim that may be made by its manufacturer, is not guaranteed or endorsed by the publisher.

